# Towards a Transferable and Cost-Effective Plant AFLP Protocol

**DOI:** 10.1371/journal.pone.0061704

**Published:** 2013-04-16

**Authors:** Marguerite Blignaut, Allan G. Ellis, Johannes J. Le Roux

**Affiliations:** 1 Centre for Invasion Biology, Stellenbosch University, Stellenbosch, Western Cape, South Africa; 2 Department of Botany and Zoology, Stellenbosch University, Stellenbosch, Western Cape, South Africa; University of Kent, United Kingdom

## Abstract

Amplified fragment length polymorphism (AFLP) is a powerful fingerprinting technique that is widely applied in ecological and population genetic studies. However, its routine use has been limited by high costs associated with the optimization of fluorescently labelled markers, especially for individual study systems. Here we develop a low-cost AFLP protocol that can be easily transferred between distantly related plant taxa. Three fluorescently labelled *Eco*RI-primers with anchors that target interspecifically conserved genomic regions were used in combination with a single non-labelled primer in our AFLP protocol. The protocol was used to genotype one gymnosperm, two monocot and three eudicot plant genera representing four invasive and four native angiosperm species (*Pinus pinaster* (Pinaceae), *Pennisetum setaceum* and *Poa annua* (Poaceae), *Lantana camara* (Verbenaceae), *Bassia diffusa* (Chenopodiaceae), *Salvia lanceolata*, *Salvia africana-lutea,* and *Salvia africana-caerulea* (Lamiaceae)). Highly polymorphic and reproducible genotypic fingerprints (between 37–144 polymorphic loci per species tested) were obtained for all taxa tested. Our single protocol was easily transferred between distantly related taxa. Measures of expected heterozygosity ranged from 0.139 to 0.196 for *P. annua* and from 0.168 to 0.272 for *L. camara* which compared well with previously published reports. In addition to ease of transferability of a single AFLP protocol, our protocol reduces costs associated with commercial kits by almost half. The use of highly conserved but abundant anchor sequences reduces the need for laborious screening for usable primers that result in polymorphic fingerprints, and appears to be the main reason for ease of transferability of our protocol between distantly related taxa.

## Introduction

Amplified Fragment Length Polymorphism (AFLP [1]) is a versatile technique for genome-wide screening of genetic diversity and can be applied to almost any organism [2]–[4]. The technique relies on detecting genetic polymorphisms through differential endonuclease restriction digestion of genomic DNA. The rapidity and large amount of data generated by this approach, as well as robustness and repeatability [3], makes AFLP analysis a commonly used tool in population genetic and ecological studies [5]. For example, a search for “amplified fragment length polymorphism” in ISI Web of Science returned more than 15000 papers, highlighting the impact of this technique since its description 17 years ago [1].

One of the biggest advantages of AFLP technology is that, unlike many other genotyping techniques, genome wide screening of genetic diversity is possible without *a priori* knowledge of genome sequences [4], [6]. The technique was originally used for constructing high density linkage maps [1], [7], but is widely applied today to estimate genetic diversity, assign parentage, determine population structure and reconstruct shallow phylogenies (reviewed by [2]). Moreover, AFLP analyses have also been adapted to assess epigenetic variation [8], [9] and differential gene expression [4], [10].

The AFLP procedure relies on complete digestion of genomic DNA by restriction enzymes, usually with a rare (e.g. *Eco*RI) and a frequent (e.g. *Mse*I) cutter. Adapters are ligated onto the digested fragments and these fragments are then amplified with a polymerase chain reaction (PCR) [1]. Conventional detection of the fragments requires radio-labelled primers and autoradiography, but advances in capillary electrophoresis allow for rapid and high throughput fluorescent or infrared detection [3], [4] and has therefore become the standard for AFLP genotyping [11].

Despite these obvious advantages, the initial costs associated with AFLP analysis can be high because large numbers of fluorescently labelled oligonucleotides often need to be screened in order to obtain sufficient polymorphism [11], [12]. Furthermore, although AFLP protocols are usually transferable between closely related taxa, or species within the same family (i.e. [13], [14]), a highly transferable AFLP protocol that can be applied to distantly related taxa is still lacking. To date, only a few attempts have been made to develop ‘universal’ AFLP protocols, and even these remain fairly taxon-specific, e.g. for sharks [15].

Here, our overall aim was to develop a time and cost efficient AFLP protocol for plants that can easily be transferred between distantly related taxa. Specifically, by targeting known conservative regions of plant genomes we hope to develop AFLP primers that will amplify fragments in a wide range of taxa. By targeting these regions we aim to develop a protocol that requires only a small number of fluorescently labelled selective oligonucleotides (in order to reduce costs) that consistently yield reproducible and highly polymorphic loci.

## Materials and Methods

### Study Species and Plant Material Collection

We collected leaf material from between 10–30 individuals representing populations of two monocots (*Pennisetum setaceum* and *Poa annua;* Poaceae), and five eudicots, *Lantana camara* (Verbenaceae), *Bassia diffusa* (Chenopodiaceae), *Salvia lanceolata*, *Salvia africana-lutea, Salvia africana-caerulea* (Lamiaceae) and *Salvi*a hybrids of unknown parentage. The two monocots as well as *L. camara* were sampled in their non-native ranges, while *B.diffusa* populations, the three *Salvia* species, and *Salvia* hybrids were collected from their native ranges. We sampled 22 plantation individuals of *Pinus pinaster* (Pinaceae) in its adventive range in South Africa. All plant material was desiccated on silica gel until further use.

All necessary collection permits were obtained for the collection of native species. The *Salvia* spp. collection was approved by Cape Nature (permit number: 0028-AAA005–00219), and the *B. diffusa* collection was approved by the Cacado Municipality district in the Eastern Cape (permit number: CRO 56/12CR). The abundant invasive species (*P. setaceum, P. annua* and *L. camara*) did not require permission for collection, and where collected along public roadsides. A permit was obtained from MTO Forestry (PTY) LTD (permit number: 65105) for the collection of *P. pinaster* in the Jonkershoek plantation, Stellenbosch, South Africa.

### DNA Extraction and AFLP Analysis

DNA was extracted either by the standard CTAB method [16] or CTAB with the addition of a 25∶24:1 phenol:chloroform:isoamylalcohol step [17]. All DNA samples were quantified using a micro-volume UV-Vis spectrophotometer (Nanodrop, Thermo Fisher Scientific, Wilmington, USA) and good quality genomic DNA (A_260/280_ ∼ 1.8 and A_260/230_∼ 2.0) was diluted to a final concentration of 100 ng/µL.

We modified the original AFLP protocol by Vos et al. [1]. For each sample, ca. 200 ng of genomic DNA was digested with 5 units of *EcoR*I (Fermentas, supplied by Inqaba Biotechnical Industries (PTY) LTD, Pretoria, South Africa) for 2 hours at 37°C in 2X Tango™ buffer (66 mM Tris-acetate, pH 7.9), 20 mM Mg-acetate, 132 mM K-acetate, 0.1 mg/ml BSA) in a 20 µL reaction volume. After *EcoR*I digestion, 5 units of *Tru*I (isoschizomer of *Mse*I; Fermentas) were added and the buffer concentration was again adjusted to 2X Tango™ buffer in a total volume of 30 µL. The reaction was incubated at 65°C (as recommended by the manufacturer) for 2 hours. A 10 µL ligation reaction mix was made up consisting of 1 unit T4 DNA ligase (Fermentas), 1X T4 DNA ligase buffer, 50 µM *Mse*I adapter and 5 µM *EcoR*I adapter (Integrated DNA Technologies [IDT], Iowa, USA; see [4] for preparation) that targets the frequent and rare cut fragments respectively, and was added directly to the digestion reaction. The digestion-ligation reaction was incubated overnight at 4°C. Following ligation, the digestion-ligation reaction mix was diluted 1∶5 with sterile distilled water and used as template for the pre-selective PCR.

Each 15 µL pre-selective PCR reaction contained 2.5 µL of the diluted digestion-ligation reaction mix, 1 µM *Mse*I+0 primer, 1 µM *EcoR*I+0 (IDT, Table 1), 1X Kapa *Taq* Readymix (contains 0.2 µM of each dNTP, 1.5 µM MgCl_2_, 0.3 unit *Taq* Polymerase, 1X Kapa Buffer A, KapaBiotech, Cape Town, South Africa supplied by Lasec SA, Cape Town, South Africa ). Pre-selective PCR amplification was done with an initial denaturing step of 94°C for 5 minutes, followed by 23 cycles consisting of denaturation at 94°C for 30 sec, annealing at 56°C for 30 sec, elongation at 72°C for 30 sec, and a final elongation step at 60°C for 30 minutes. Successful amplification was confirmed by running 5 µL of the PCR product on a 1% agarose gel and observing a smear between 100 and 500 bp.

**Table 1:** Oligonucleotide sequences for all the primers required for the standardized AFLP protocol.

Primer name	Sequence (5′–3′)	Length (bp)	Label	Final concentration (µM)
***EcoR*** **I-adapter forward**	CTCGTAGACTGCGTACC	17	None	5
***EcoR*** **I-adapter reverse**	AATTGGTACGCAGTCTAC	18	None	5
***Mse*** **I-adapter forward**	GACGATGAGTCCTGAG	16	None	50
***Mse*** **I-adapter reverse**	CTACTCAGGACTCAT	15	none	50
***EcoR*** **I+0**	GACTGCGTACCAATTC	16	none	1
***Mse*** **I+0**	GATGAGTCCTGAGTAA	16	none	1
***Mse*** **I-CTT**	GATGAGTCCTGAGTAACTT	19	none	1
***EcoR*** **I-ATG**	GACTGCGTACCAATTCATG	19	6-Hex™ (IDT)	0.25
***EcoR*** **I-CAT**	GACTGCGTACCAATTCCAT	19	Fam™ (IDT)	0.25
***EcoR*** **I-AAT**	GACTGCGTACCAATTCAAT	19	Ned™ (Applied Biosystems)	0.25

Following successful amplification, pre-selective PCR products were diluted with sterile distilled PCR-grade water (1∶19 dilution) of which 5 µL was used as template for selective PCR amplification. Each 20 µL selective PCR reaction contained 0.25 µM of fluorescently-labelled *EcoR*I+NNN (see Table 1 for anchor and label) and 1 µM unlabelled *Mse*I+CTT (IDT; Table 1), and 1X Kapa *Taq* Readymix. PCR reactions were done without a step-down PCR step [1], [4] following pre-selective PCR conditions described above but with 30 repeat cycles.

After amplification, 5 µL of each fluorescently-labelled PCR product was mixed for each DNA sample and purified using the NucleoFast Purification System (Machery-Nagel Gmbh and Co.kG, Düren, Germany). Electrophoresis was performed on the 3130×l DNA Analyser (Applied Biosystems, California, USA) with the ROX500 size standard (Applied Biosystems).

Automated fragment size calling and scoring was performed with Genemarker Version 2.2.0 (SoftGenetics, LLC, CA, USA) with the manufacturer’s default settings. The presence or absence of all fragments was confirmed manually since intensity differences between samples might result in false absences. All individuals within each species were scored in a single session to avoid manual scoring artefacts and errors. Only loci (fragments) between 100 and 450 bp were scored to decrease the possible detection of co-migrating fragments, i.e. size homoplasy [18].

#### Genetic diversity

Locus-specific variability was measured with the polymorphic information content (PIC) for dominant markers [19].

For each species we generated a binary presence-absence data matrix. From this we calculated the total number of loci generated per primer pair as well as the percentage of polymorphic loci for each taxon in GenAlEx version 6.4 [20]. Expected heterozygosity (H_E_), under the assumption of Hardy-Weinberg equilibrium, was also calculated in GenAlEx version 6.4 [21].

#### Reproducibility and average peak intensity

Reproducibility of obtained AFLP banding profiles was assessed by repeating all experimental steps on at least 10% of all individuals genotyped per population [22]. The *Salvia* spp. were analysed together and a single error rate was calculated for all the samples analysed. Error rates were determined as the percentage of loci that were mismatched between the replicate pairs [23]. Furthermore, the average peak intensity was calculated across all scored loci and compared across species for significant differences using a Kruskal-Wallis test in the R statistical environment [24]. Dunn’s post hoc test was performed to compare the difference in rank sum for each species in Graphpad Prism V5.01 (GraphPad Software, Inc.).

## Results

### Genetic Diversity

The usefulness of population genetic markers, for example, in parentage assignment and linkage studies, is measured by how informative they are (polymorphism information content (PIC) *sensu* [25], [26]. Even though these applications were not explored here, we determined that the PIC of each primer pair was both comparable between species and between markers (Table 2). Overall, our PIC values ranged from 0.003–0.379, where markers with PIC ≥0.3 are considered of high discriminatory value [19].

**Table 2:** Summary data for *Bassia diffusa, Lantana camara, Pennisetum setaceum, Poa annua, Salvia* sp. hybrids, *Salvia lanceolata*, *Salvia africana-lutea,* and *Salvia africana-caerulea* and *Pinus pinaster*.

Diversity estimates across all primer combinations						
Species name	Populationsize (*n)*	Total numberof bands	Percentage ofpolymorphic bands	Mean expectedheterozygosity (H*_e_*)	Average Polymorphicinformation content (PIC)	Scoringerror rate
***Bassia diffusa***	6	144	100%	0.261	0.29	3.82%
***Lantana camara***	19	52	71.15%	0.272	0.17	3.84%
***Pennisetum setaceum***	25	37	8.11%	0.026	0.01	1.35%
***Poa annua***	19	80	41.25%	0.167	0.11	3.75%
***Salvia hybrids***	5	88	48.86%	0.194	0.2	2.10%
***Salvia africana-caerulea***	17	95	80.00%	0.259	0.21	2.10%
***Salvia africana-lutea***	12	95	68.42%	0.257	0.21	2.10%
***Salvia lanceolata***	12	95	78.95%	0.277	0.24	2.10%
***Pinus pinaster***	10	53	41.51%	0.147	0.11	2.83%
**Primer combination: ** ***EcoR*** **I-CAT-Fam™ and Mse+CTT**						
**Species name**	**Population** **size (** ***n)***	**Total number** **of bands**	**Percentage of** **polymorphic bands**	**Mean expected** **heterozygosity (H** ***_e_*** **)**	**Average Polymorphic** **information content (PIC)**	**Scoring** **error rate**
***Bassia diffusa***	18	70	100%	0.261	0.38	1.90%
***Lantana camara***	27	24	87.50%	0.304	0.19	2.78%
***Pennisetum setaceum***	30	19	5.26%	0.016	0	0.00%
***Poa annua***	19	40	32.50%	0.139	0.1	2.50%
***Salvia hybrids***	12	41	60.98%	0.236	0.19	0.81%
***Salvia africana-caerulea***	15	41	68.29%	0.258	0.18	0.81%
***Salvia africana-lutea***	12	41	68.29%	0.226	0.2	0.81%
***Salvia lanceolata***	12	41	75.61%	0.176	0.25	0.81%
***Pinus pinaster***	22	18	44.44%	0.131	0.07	1.85%
**Primer combination: ** ***EcoR*** **I-ATG-Hex™ and Mse+CTT**						
**Species name**	**Population** **size (** ***n)***	**Total number** **of bands**	**Percentage of** **polymorphic bands**	**Average expected** **heterozygosity (H** ***_e_*** **)**	**Average Polymorphic** **information content (PIC)**	**Scoring** **error rate**
***Bassia diffusa***	17	48	100%	0.257	0.37	3.47%
***Lantana camara***	26	12	83.33%	0.333	0.17	2.56%
***Pennisetum setaceum***	30	10	60.00%	0.221	0.07	5.00%
***Poa annua***	19	21	47.62%	0.194	0.14	3.17%
***Salvia hybrids***	13	34	72.73%	0.399	0.27	0.9%%
***Salvia africana-caerulea***	15	34	84.85%	0.227	0.2	0.9%%
***Salvia africana-lutea***	12	34	69.70%	0.303	0.22	0.9%%
***Salvia lanceolata***	12	34	81.82%	0.281	0.23	0.9%%
***Pinus pinaster***	22	19	57.89%	0.191	0.1	2.63%
**Primer combination: ** ***EcoR*** **I-AAT-Ned™ and Mse+CTT**						
**Species name**	**Population** **size (** ***n)***	**Total number** **of bands**	**Percentage of** **polymorphic bands**	**Average expected** **heterozygosity (H** ***_e_*** **)**	**Average Polymorphic** **information content (PIC)**	**Scoring** **error rate**
***Bassia diffusa***	6	26	100%	0.289	0.36	3.84%
***Lantana camara***	19	15	86.67%	0.373	0.27	3.33%
***Pennisetum setaceum***	30	8	12.50%	0.037	0.01	0.00%
***Poa annua***	17	19	52.63%	0.196	0.11	2.63%
***Salvia hybrids***	5	22	59.09%	0.223	0.2	4.54%
***Salvia africana-caerulea***	12	22	81.82%	0.248	0.2	4.54%
***Salvia africana-lutea***	12	22	63.64%	0.234	0.21	4.54%
***Salvia lanceolata***	12	22	77.27%	0.26	0.23	4.54%
***Pinus pinaster***	10	16	31.25%	0.118	0.07	3.25%

The total number of scored loci and percentage of polymorphic loci, average heterozygosity (*H_e_*), polymorphic information content (PIC) and the scoring error rate, are shown for combined and individual primer pair combinations.

**Figure 1:**
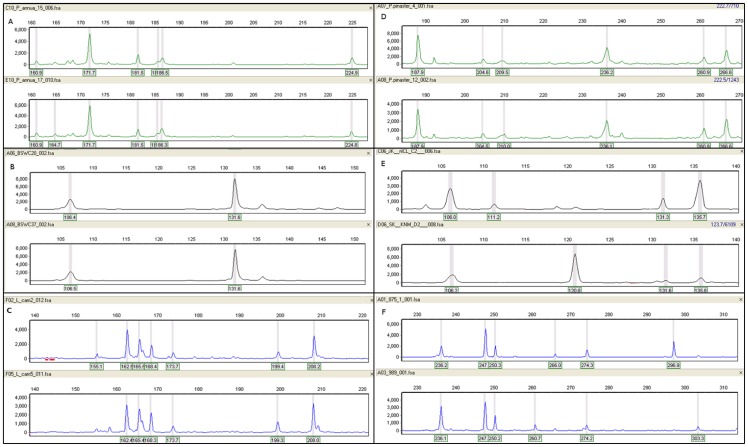
Typical fingerprint profiles generated with the primer pairs: *EcoR*I-ATG- Hex™+Mse-CTT for A) two *Poa annua* samples and D) 2 *Pinus pinaster* samples; B) *EcoR*I-AAT-Ned+Mse-CTT for two *Pennisetum setaceum* and E) two *Bassia diffusa* samples samples; and *EcoR*I-CAT-FAM+Mse-CTT for C) two *Lantana camara* samples, and F) two *Salvia* spp. samples.

Our AFLP protocol yielded highly polymorphic loci and was successfully transferred between the eight species included here (data summarised in Table 2) and generated a minimum of 5.26% polymorphic loci for *P. setaceum* (for EcoRI-AAT NED™), and a maximum of 100% polymorphic loci for *B. diffusa* (for all three labelled primers). The total number of loci generated for *P. setaceum* (37 of which 8.11% were polymorphic overall) were the lowest, whilst we amplified a total of 144 loci in *B. diffusa* A previous AFLP study on *P. annua* reported 60% polymorphic loci out of the 226 loci analysed [27]. We amplified a total of 80 loci for *P. annua* of which 41.25% were polymorphic. *Lantana camara* and *P. pinaster* had an intermediate number of 52 loci (of which 71.15% were polymorphic) and 53 loci (41.51% polymorphic), respectively. The three Salvia species (*S. africana-lutea, S. africana-caerulea* and *S. lanceolata*) yielded 95 loci each of which 68.42%, 80.0% and 78.95% were polymorphic, respectively. The *Salvia* hybrids (of unknown parentage) yielded 99 loci of which 48.86% were polymorphic. This is the first report of AFLP fingerprints for *B. diffusa*, *P. setaceum* and the three *Salvia* species. The number of loci generated for the native *B. diffusa* and *Salvia* spp., are within the range of 100–150 loci for which can be used for fine-scale spatial genetic structure assessment, although the use of much larger numbers of loci (up to 250) is suggested [28]. Based on this criterion, the low and intermediate number of loci generated for *P. setaceum*, *L. camara*, *P. annua* and *P. pinaster* might be insufficient to reveal the true fine-scale population genetic structure. Typical fingerprints generated for the eight species are shown in Figure 1.

We also determined expected heterozygosity (*H_E_*), which is a measure of within-population gene diversity and is equivalent to Nei’s unbiased gene diversity (*H_S_*), as adapted for dominant markers under the assumptions of Hardy-Weinberg equilibrium and the Lynch-Milligan model [21]. Here, the overall expected heterozygosity (*H_E_* = 0.272) for the combined *L. camara* loci was the highest observed out of the eight species, but is lower than the range (*H_E_*: 0.336–0.848) previously reported for this species based on co-dominant microsatellites [29]. It does however fall within recently reported Nei’s gene diversity values (0.023–0.293) for *L. camara* [30]. For *P. annua*, our heterozygosity estimate (*H_E_* = 0.167) fell within the previously published range based on estimates of Nei’s unbiased gene diversity (*H_S_* = 0.152 based on AFLP markers [31] and *H_S_* = 0.245 using RAPD markers [32]). The expected heterozygosity for *P. pinaster* (*H_E_* = 0.147) compared well with previously published results based on 122 loci for two populations(*H_S_* = 0.159 and 0.162 respectively [33]). The average expected heterozygosity for the three *Salvia* species ranged between 0.259–0.277 (in the order listed in Table 2), while the *Salvia* hybrids showed the lowest within population diversity (*H_E_* = 0.194). The low combined expected heterozygosity for all the *P. setaceum* loci (*H_E_* = 0.03) is not surprising given previous reports [34], [35] that showed no genetic variation within or among populations of *P. setaceum* based on dominant ISSR markers, microsatellites and DNA sequencing data.

### Reproducibility and Average Peak Intensity

We assessed data quality of our protocol by determining the error-rate and reproducibility of our datasets. The suggested and generally acceptable error rate for AFLP data ranges between 2–5% [12]. Here, for samples that were genotyped twice, we found the lowest average error rate (calculated with the lowest number of repeats for a marker) across all markers for *P. setaceum* (1.35%), and the highest error rate for *B. diffusa* (3.82%), and *L. camara* (2.84%), with intermediate values for *P. annua* (3.75%), *Salvia* spp. (2.1%), and *P pinaster* (2.83%). Error rates were never greater than 5% indicating that our protocol is highly reproducible across a wide variety of species representing different plant families.

**Figure 2:**
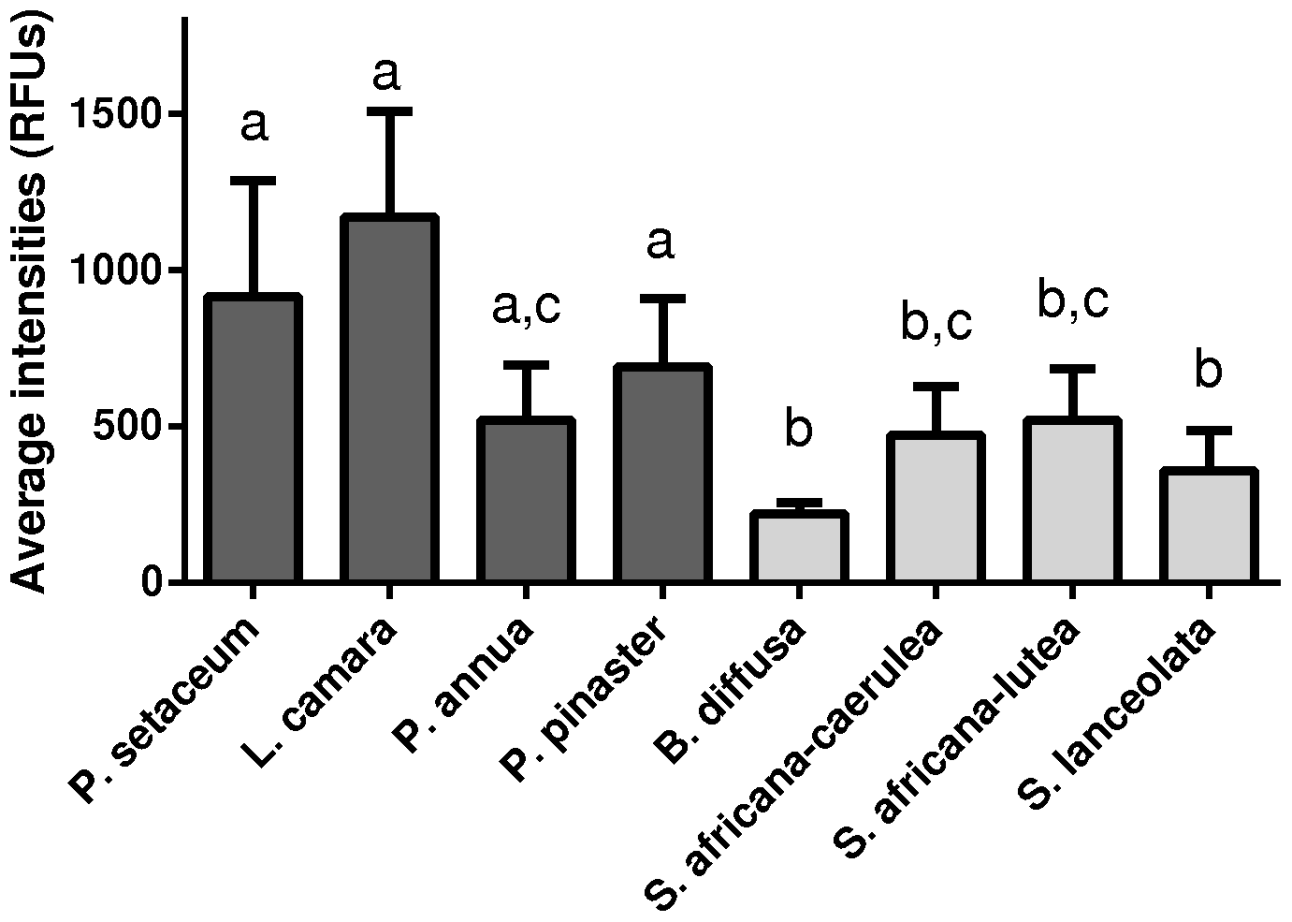
A comparison of mean fluorescence intensity (±standard error, SE) between the species sampled. No significant differences were found between the invasive species (dark grey). The four native species and *Salvia* hybrids (shown in light grey) differed significantly from all of the invasive species (with exceptions), but not from each other (Kruskal-Wallis Chi squared = 125.9, df = 8, *P*<0.0001). Samples with the same letters do not differ significantly, whilst different letters indicate significant differences between the species and were determined with Dunn’s post hoc tests.

In order to determine and compare the overall amplification success we compared peak intensity (a measure of data quality) between the different species and found that *B. diffusa* and the *Salvia* spp. profiles had significantly lower fluorescence intensities than the other species (Kruskal-Wallis Chi squared = 125.9, df = 8, *P*<0.0001, Figure 2). Compared to all other taxa, more loci were generated for *B. diffusa* and the *Salvia* sp., which likely resulted in overall reduced fluorescence.

## Discussion

Although capillary electrophoresis has become the standard for AFLP genotyping, the costs associated with screening numerous fluorescent primers for individual taxa remain prohibitively expensive. Here we describe a modified AFLP protocol that can easily and successfully be transferred across a wide range of closely and distantly related plant taxa with high repeatability.

We tested the technique on eight species from five different plant families, representing monocots (Poaceae), eudicots (Verbenaceae, Chenopodiaceae and Lamiaceae), and one gymnosperm (Pinaceae). Our sampling encompassed taxa exhibiting variation in life history traits such as growth form and geographic range size which tend to influence the amount of genetic variation within species [36]. Also, some taxa were sampled from their introduced ranges and would thus be expected to have reduced genetic variability [37], [38], whereas others were natives. Variation in ploidy and genome size can also affect the numbers of bands observed and the quality of AFLP profiles [39], we included angiosperms with 1C genome contents that ranged from 1.4 ρg (*P. setaceum,* triploid) to 2.88 ρg (*P. annua,* tetraploid), as well as a gymnosperm (*P. pinaster,* diploid) with a 1C content of 28.90 ρg. Our protocol yielded polymorphic and highly reproducible AFLP fingerprints across all these taxa.

As expected for high quality AFLP markers, all our markers generated clear scorable genotypic fingerprints which were spread evenly along profiles for all species included [28]. The three primer pairs (*EcoR*I-labelled primers) used here were designed with three base pair anchors that target specific and conserved regions within most plant genomes, similar to sequence-specific amplified polymorphisms (SSAP, [40]). The *EcoR*I-ATG anchor (which also has the highest PIC value of 0.196), was designed to target gene transcription initiation regions (ATG-) which are conserved motifs (AUG) found throughout the genome within coding, intronic and intergenic regions [41]. The intermediately variable of the three primers tested here (PIC = 0.184) targets the TATA-box region (TATAA-motif) upstream from the transcription initiation motif (ATG), which is a highly conserved but rare region that has been recorded in all species investigated to date [42]. Our primer combination with the lowest PIC value (0.174) targets the more common –CAT gene motif not associated with any conserved region.

Screening for primer pairs that create sufficiently polymorphic loci requires extensive technical expertise and is expensive [11]. Although there are many commercial kits available for AFLP analysis, these also require extensive screening of different primer pairs to obtain sufficiently polymorphic loci and tend to be done in a species-specific manner. Compared to a leading commercial kit, our protocol costs approximatly half (∼7 $ US vs. 15 $ US) to perform for three labelled primer pairs per sample. It should also be noted that our protocol worked for *B. diffusa* for which fragment amplification failed after numerous attempts using a commercial kit. Although the number of loci for *P. setaceum*, *P. pinaster* and *L. camara* might not be sufficient for fine-scale genetic structure analysis (although the latter diversity indices compared well to published results [29], [30], [33]–[35]) these labelled primers were designed to target specific genome wide regions found in all living organisms. It should thus be possible to increase the number of fragments by merely adding another unlabelled Mse-NNN primer to increase the number of primer pairs at nominal cost.

In summary, we developed a cost- and time-effective AFLP protocol for large-scale high-throughput data generation that only requires three selective fluorescently-labelled primers, eliminating the need for extensive screening of suitable primer combinations, while simultaneously providing highly polymorphic and informative loci that are reproducible. Moreover, our protocol is readily transferable between distantly related plant taxa, further eliminating tedious optimization steps normally required when transferring AFLPs to new taxa. We speculate that by targeting additional regions that are known to be conserved throughout genomes as anchors for PCR primers, that our protocol could be easily adapted across all forms of life.
